# Mechanisms and therapeutic strategies linking mesenchymal stem cells senescence to osteoporosis

**DOI:** 10.3389/fendo.2025.1625806

**Published:** 2025-07-21

**Authors:** Yashuang Tong, Yulin Tu, Jingying Wang, Xiuyu Liu, Qian Su, Yanghao Wang, Weizhou Wang

**Affiliations:** ^1^ Department of Orthopedics, The First Affiliated Hospital of Kunming Medical University, Kunming, Yunnan, China; ^2^ First School of Clinical Medicine, The First Affiliated Hospital of Kunming Medical University, Kunming, Yunnan, China; ^3^ Department of Pathology, The First Affiliated Hospital of Kunming Medical University, Kunming, Yunnan, China

**Keywords:** mesenchymal stem cells, aging, osteoporosis cellular senescence, bone regeneration, senescence-associated secretory phenotype, oxidative Stress

## Abstract

Osteoporosis is a common age-related bone metabolic disorder that significantly affects skeletal health, especially in aging populations. With global demographic shifts, the rising prevalence and disability burden of osteoporosis has placed increasing pressure on healthcare systems, making it a key area of research. A crucial factor in osteoporotic progression is the aging of mesenchymal stem cells (MSCs), which weakens bone regeneration through multiple mechanisms, including reduced osteogenic differentiation, heightened oxidative stress, chronic inflammation, and disrupted bone homeostasis. This review explores the intricate relationship between MSCs aging and osteoporosis development, focusing on key processes such as cell cycle arrest, telomere shortening, epigenetic changes, and osteogenic marker expression dysregulation. We also examine potential therapeutic strategies aimed at alleviating MSCs aging, including stem cell-based treatments, senolytic agents, inhibitors targeting the senescence-associated secretory phenotype, and biomaterial-assisted approaches such as extracellular vesicles and stimuli-responsive hydrogels. This review aims to provide insights into developing precise therapeutic strategies to restore MSCs function and slow bone loss. Furthermore, we discuss interdisciplinary approaches that link molecular mechanisms to practical applications, offering a broader perspective on addressing osteoporosis in aging societies.

## 1 Introduction

Aging is an irreversible physiological process in living organisms, characterized by a gradual decline in physiological functions, which leads to tissue damage and dysfunction. Aging is not only a natural biological phenomenon but also a fundamental cause of many chronic degenerative diseases, including cancer, diabetes, osteoporosis, and Alzheimer’s disease (1). At the macroscopic level, aging is characterized by a progressive decline in cellular proliferation and repair capacity, increasing susceptibility to disease and physiological deterioration. The mechanisms of aging are multifaceted, among which cellular senescence serves as a central contributor (2). Hallmark features of cellular senescence include reduced proliferative capacity, metabolic dysregulation, and heightened inflammatory responses. Notably, stem cell senescence, as a pivotal aspect of aging, exerts a more profound impact on the entire organism than other cell types. Stem cell senescence not only limits tissue regeneration and repair but also exacerbates multiple pathological changes associated with aging. Consequently, delaying stem cell senescence has emerged as a crucial research focus in the field of anti-aging.

Cellular senescence is closely linked to various age-related diseases, particularly playing a key role in bone metabolic disorders such as osteoporosis. With increasing age, the proliferative capacity of mesenchymal stem cells (MSCs) declines, especially the osteogenic differentiation potential of bone marrow-derived mesenchymal stem cells (BMSCs), leading to decreased bone density and deterioration of bone microarchitecture. The aging of MSCs directly results in impaired bone tissue regeneration, which constitutes one of the primary pathological mechanisms of primary osteoporosis (3, 4). Moreover, MSCs senescence is accompanied by an increased secretion of pro-inflammatory factors, further exacerbating osteoporosis progression (5). Therefore, investigating the relationship between MSCs senescence and osteoporosis not only provides deeper insights into the pathophysiology of osteoporosis but also offers a novel theoretical foundation for its prevention and treatment. This review comprehensively examines the relationship between MSCs senescence and osteoporosis, elaborates on the specific mechanisms underlying MSCs aging in osteoporosis pathogenesis, and summarizes current therapeutic strategies and the application of emerging pharmacological interventions. The aim is to provide a more comprehensive perspective and guidance for future research and therapeutic advancements in osteoporosis.

## 2 The mechanism and influencing factors of stem cell aging exacerbating osteoporosis

Stem cell senescence serves as a pivotal driver of osteoporotic pathogenesis. During cellular aging, the expression of osteogenic markers, such as Runx2 and Osterix, decreases (6, 7), and coincides with elevated oxidative stress (8), inflammatory microenvironment imbalance, and bone marrow microcirculatory dysfunction. These synergistic perturbations collectively suppress osteoblast differentiation and bone formation, thereby perturbing bone remodeling equilibrium and accelerating osteoporotic progression.

### 2.1 Stem Cell Senescence Suppresses Osteogenic Markers and Accelerates Osteoporosis

Stem cell senescence critically impairs osteogenic marker expression and directly drives osteoporotic progression. MSCs, a class of pluripotent progenitor cells, are widely distributed in bone marrow (9), adipose tissue (10), umbilical cord blood (11), and dental pulp (12), with the capacity to differentiate into osteoblasts, chondrocytes, and adipocytes, thereby holding significant potential in regenerative medicine and tissue engineering (13). Under the regulation of bone morphogenetic protein (BMP) and Wnt/β-catenin signaling, MSCs sequentially differentiate into osteoprogenitor cells (14, 15), mature osteoblasts, and ultimately functional osteocytes (16). Osteoblasts, the principal synthetic cells of the bone matrix, are regulated by core osteogenic markers including Runx2 (17), Osterix (7, 17), osteocalcin, and alkaline phosphatase (18, 19). However, senescent MSCs exhibit marked downregulation of these key factors, accompanied by diminished osteogenic differentiation capacity (18). Accumulating evidence demonstrates that both mRNA and protein levels of Runx2 and Osterix are reduced in senescent MSCs, correlating with alterations in intracellular signalling pathways (20). When the activity of BMP and Wnt/β-catenin signalling pathways is reduced, the production and quality of related proteins (e.g., Smad proteins, Wnt proteins) are reduced, which directly affects the activation of osteogenic genes, leading to insufficient synthesis and mineralisation of bone matrix, thus accelerating the development of osteoporosis. Collectively, MSCs senescence exacerbates osteoporosis via suppression of osteogenic differentiation, highlighting therapeutic opportunities to target this axis.

### 2.2 The interaction between stem cell aging and oxidative stress exacerbates osteoporosis, and regulating antioxidants can improve the condition.

The crosstalk between stem cell senescence and oxidative stress represents a pivotal contributor to osteoporotic pathogenesis. Oxidative stress arises from an imbalance between intracellular reactive oxygen species (ROS) production and antioxidant defense systems, culminating in macromolecular damage and cellular dysfunction. Mechanistically, excessive ROS activate DNA repair mechanisms, upregulate senescence-associated molecules (e.g., p53, p21), and induce stem cell senescence by triggering DNA damage, protein denaturation, and cell membrane disruption, ultimately promoting apoptosis (21). Furthermore, ROS amplifies local inflammatory responses through enhanced secretion of pro-inflammatory cytokines such as interleukin-6 (IL-6) and tumor necrosis factor-α (TNF-α), further promotion of stem cell senescence (22). Senescent stem cells exhibit markedly reduced antioxidant enzyme activity, with superoxide dismutase (23), catalase (24), and glutathione peroxidase (25) levels declining significantly compared to their non-senescent counterparts. This enzymatic impairment compromises ROS scavenging capacity, leading to intracellular oxidative stress accumulation, which exacerbates oxidative damage and functional degradation (25, 26). Moreover, recent studies suggest that in addition to MSCs, osteocyte senescence also contributes to bone homeostasis dysregulation. According to Frost’s mechanostat theory (27), osteocytes sense mechanical loading and regulate bone architecture to maintain mechanical integrity. However, aging leads to osteocyte senescence, which impairs their mechanosensitivity and downstream signaling pathways. This results in reduced bone adaptability to mechanical stress, decreased bone strength, and increased fracture risk (28). Consequently, in the pathological process of osteoporosis, a vicious circle is formed between oxidative stress and stem cell senescence: oxidative stress accelerates stem cell senescence by damaging key intracellular molecules and cellular structures; whereas senescent stem cells accelerate the senescence process by decreasing the antioxidant capacity and causing further accumulation of ROS. This vicious circle significantly reduces the self-renewal capacity and multidirectional differentiation potential of stem cells, affecting the regenerative capacity of tissues (29, 30). Therapeutic targeting of this axis demonstrates translational potential. By reducing ROS accumulation and restoring stem cell antioxidant capacity, cellular senescence can be attenuated, thereby promoting bone health. Current evidence indicates that antioxidant supplementation or specific antioxidant therapies significantly reduce oxidative stress levels and ameliorate clinical manifestations of osteoporosis. Thus, developing novel strategies to disrupt the senescence-oxidative stress interaction offers critical insights for osteoporosis prevention and treatment.

### 2.3 Inflammatory microenvironment accelerates the aging of BMSCs, enhances the risk of osteoporosis, and regulating inflammation can slow down the condition.

The relationship between the inflammatory microenvironment and the aging of BMSCs has become a prominent research focus in recent years. The inflammatory environment plays a pivotal role in accelerating BMSCs senescence, thereby promoting the development of osteoporosis. Inflammatory factors, including cytokines (e.g., TNF-α, interleukin-1, IL-6), chemokines (e.g., monocyte chemoattractant protein-1, CXCL-8), and prostaglandins (e.g., PGE_2_) (32–34, 216), contribute to the inflammatory response through distinct mechanisms. Cytokines regulate immune cell activity to amplify inflammation, chemokines recruit leukocytes to sustain inflammatory responses, and prostaglandins act as lipid signaling molecules involved in inflammation and nociception. Anti-inflammatory factors such as interleukin-10 and transforming growth factor-β counteract inflammation by suppressing pro-inflammatory cytokine production and maintaining inflammatory homeostasis. In aging or disease states, chronic inflammation accelerates BMSCs senescence, exacerbating osteoporosis. Senescent MSCs secrete pro-inflammatory factors (e.g., TNF-α, IL-6) that perpetuate local inflammation while suppressing their proliferative and osteogenic potential, thereby impairing bone repair and remodeling (36, 217). The inflammatory response promotes MSCs senescence through two primary mechanisms: on the one hand, persistent secretion of inflammatory factors elevates ROS levels, impairing stem cell function and promoting senescence (37); on the other hand, the inflammatory microenvironment inhibits osteogenesis and enhances bone resorption by modulating interactions between MSCs and osteoblasts, bone-resorbing cells (38). This process not only compromises bone health but also disrupts bone marrow microenvironment homeostasis, exacerbating osteoporotic manifestations (39). Additionally, senescent MSCs exhibit a senescence-associated secretory phenotype (SASP), characterized by increased inflammatory burden, reduced osteoblast function, and accelerated bone density loss (40, 41). Thus, the inflammatory microenvironment not only accelerates BMSCs aging but also directly elevates osteoporosis risk.

Modulating the inflammatory response represents a promising therapeutic strategy. By inhibiting pro-inflammatory factors (e.g., TNF-α, IL-6) or activating anti-inflammatory pathways (e.g., Transforming Growth Factor-beta, interleukin-10), it is possible to attenuate MSCs senescence and restore their differentiation and self-renewal capacity (42, 43). Anti-inflammatory treatments reduce SASP production, suppress bone marrow inflammation, and improve bone mineral density (BMD) while lowering fracture risk (44, 45). These findings highlight the potential of targeting the inflammatory microenvironment to alleviate osteoporosis and delay BMSCs aging, providing a theoretical foundation and practical framework for clinical osteoporosis management.

### 2.4 Impaired bone marrow microcirculation function is a key mechanism that promotes stem cell aging and the progression of osteoporosis.

Alterations in bone marrow microcirculation, particularly reduced vascularization, are critical contributors to stem cell senescence and osteoporosis (46). Bone marrow microcirculation comprises a network of microvessels, including micro arterioles, microbes, and capillaries, which supply oxygen and nutrients to bone marrow stem cells while removing metabolic waste, thereby maintaining local environmental stability (47). However, with aging or in osteoporotic conditions, both the quantity and quality of blood vessels decline, leading to diminished microcirculatory function, local hypoxia, insufficient nutrient supply, and reduced angiogenic capacity. These changes directly impair stem cell function, particularly in BMSCs (48). Specifically, senescent BMSCs exhibit reduced self-renewal and differentiation capacities and are more prone to entering a senescent state under hypoxic and nutrient-deficient conditions (49). Vascular endothelial growth factor (VEGF) is a key regulator of bone marrow microcirculation and angiogenesis (50). VEGF promotes neovascularization by stimulating endothelial cell proliferation and migration, ensuring adequate nutrient and oxygen supply to BMSCs (51). However, VEGF levels decline with aging, accompanied by microcirculatory dysfunction, leading to BMSCs functional decline and exacerbating osteoporotic progression (52). Mechanistically, VEGF binds to its receptor VEGFR, activating downstream signaling pathways such as PI3K/Akt and MAPK, which regulate cell proliferation, survival, and angiogenesis (53, 54). For instance, PI3K/Akt activation promotes cell survival and inhibits apoptosis, while also enhancing cell proliferation through the regulation of cell cycle-related factors (e.g., Cyclin D1) (55). Similarly, MAPK activation further modulates cell proliferation, migration, and angiogenesis. The decline in VEGF expression and function weakens anti-apoptotic effects, reduces angiogenesis, and deteriorates microcirculation, thereby accelerating osteoporosis (56, 57). Therefore, maintaining bone marrow microcirculation function, enhancing angiogenesis, and improving microcirculatory efficiency represent promising strategies to mitigate stem cell senescence and osteoporotic progression, ultimately promoting bone health.

## 3 Mechanisms and modulators of MSCs aging

### 3.1 Intrinsic drivers of stem cell senescence

#### 3.1.1 Cell cycle dysregulation and stem cell senescence

The stem cell cycle, encompassing the G1, S, G2, and M phases, is fundamental to cell proliferation, repair, and differentiation. Regulation of the stem cell cycle involves cyclins, cyclin-dependent kinase (CDK), and CDK inhibitors (e.g., p21, p15, p53) (58, 59). Among these phases, G1 and G2 are particularly critical in cellular senescence, as DNA damage during these phases can induce cell cycle arrest and impair cellular function.

During the G1 phase, cells synthesize proteins necessary for DNA replication and continued growth. Stem cell aging is often associated with G1 phase arrest, which inhibits cell proliferation (60). DNA damage accumulates in stem cells with age due to endogenous metabolic stress and exogenous factors (e.g., ultraviolet light, chemical toxins), and is detected and repaired through the DNA damage response pathway (61). DNA damage response activation primarily depends on p53 (62), p21, and p15 (59), which inhibit CDK activity or increase CDK inhibitor levels to arrest the cell cycle in the G1 phase (63). Specifically, p53 upregulates p21 to prevent cells from entering the S phase, ensuring DNA repair; if repair fails, cells undergo senescence or apoptosis (64, 65). p15 restricts cell proliferation and promotes senescence by inhibiting CDK4/6 (66). Additionally, p21 maintains the non-phosphorylated state of Rb proteins, inhibits E2F activity, and impairs MSCs self-renewal and differentiation (59). These changes deplete the MSCs pool, reduce osteogenic capacity, and accelerate osteoporotic progression.

In the G2 phase, cells ensure accurate genetic material transmission through DNA damage detection, a critical quality control checkpoint. Senescent MSCs exhibit impaired G2 phase regulation, preventing entry into the M phase and compromising regenerative capacity. Unrepaired DNA damage (e.g., double-strand breaks) triggers G2 phase arrest, which typically occurs shortly after DNA damage and persists if repair fails. Activation of the p53 pathway induces p21 expression and inhibits CDK1, preventing M phase entry and maintaining G2 phase arrest (67, 68). Chk1 and Chk2 further inhibit Cdc25 phosphatase activity, downregulating CDK1 and preventing M phase transition (69). Persistent DNA damage response activation due to DNA damage accumulation leads to permanent G2 phase arrest, reducing MSCs numbers and tissue repair capacity, ultimately contributing to osteoporosis (70, 71).

**Figure 1:**
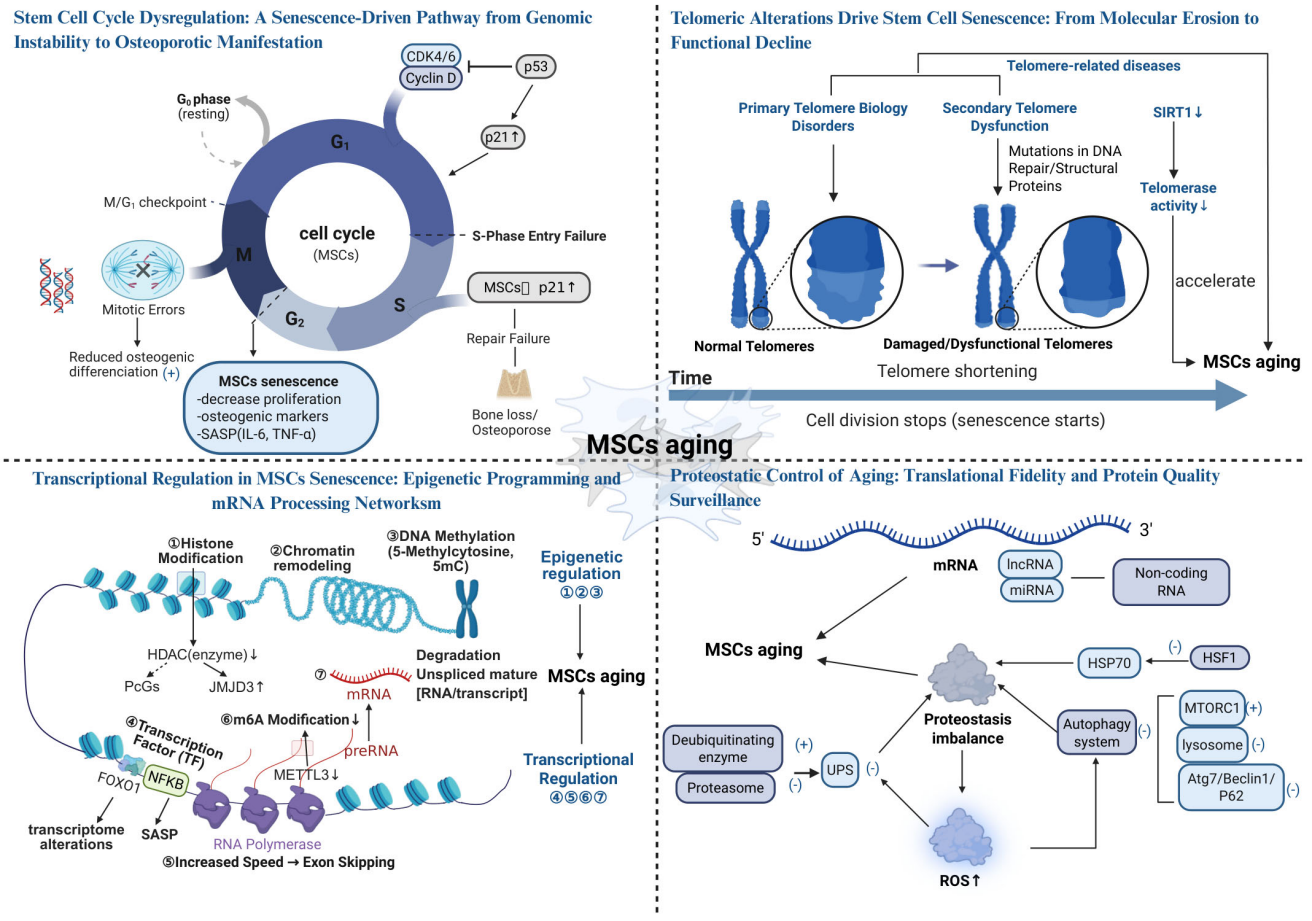
The Aging Mechanism of Stem Cells (Created in https://BioRender.com).

The S phase involves DNA synthesis and replication, while the M phase encompasses cell division. During aging, the S phase is frequently disrupted by DNA damage or oxidative stress. When damage exceeds stem cell repair capacity, repair failure occurs, upregulating proteins such as p52 and p21. These proteins regulate cell cycle checkpoints, halting cells in the G1 or G2 phase to prevent replication of damaged DNA (72, 73). In the M phase, senescent stem cells often exhibit division errors (e.g., chromosomal abnormalities or unequal division) due to unrepaired DNA damage, resulting in M phase arrest or aberrant division (74, 75). In summary, the S and M phases regulate cell proliferation and differentiation through DNA damage and abnormal cell division, respectively. Therefore, cell cycle impairment is a central mechanism underlying stem cell senescence and osteoporosis. Strategies to repair DNA damage and modulate key regulators such as p53, p21, and p15 may offer novel approaches to slowing osteoporotic progression (57). Stem cell cycle dysregulation is presented in **Figure 1**.

#### 3.1.2 The impact of telomere alterations on cellular aging

Telomeres are protective structures at chromosome ends, composed of TTAGGG repeat sequences and associated proteins that form t-loops to prevent chromosomal misrecognition or degradation (76, 77). Telomere shortening and dysfunction are critical contributors to cellular senescence and related diseases (78, 79). Due to the inherent limitations of DNA replication, tens of telomere base pairs are lost with each cell cycle (80, 81), leading to progressive telomere attrition. Although human embryonic stem cells and tumor cells can delay aging through telomerase expression (82, 83), most human somatic cells lack active telomerase and cannot maintain telomere length (84). This results in critically short and dysfunctional telomeres in some cells (85). Extremely short telomeres trigger DNA damage signaling and telomere dysfunction (86, 87, 213), leading to cell cycle arrest and increased susceptibility to DNA damage as telomeres shorten (89, 90). Dysfunctional telomeres also induce cellular senescence, particularly affecting rapidly dividing or regenerating tissues. Additionally, SIRT1 plays a key role in telomerase regulation, and its inhibition reduces telomerase activity, accelerating MSCs senescence and contributing to osteoporosis (91, 92).

Telomere-associated disorders, which promote stem cell senescence, are classified into two categories: primary and secondary telomere diseases (86, 93). Primary telomere diseases result from mutations in telomerase maintenance genes (e.g., DKC1, hTERC, or hTERT), impairing telomerase activity, accelerating telomere shortening, and promoting MSCs senescence (89). This leads to a reduction in pre-osteoblast differentiation, contributing to osteoporosis. Secondary telomere diseases arise from mutations in DNA repair or structural proteins, with environmental factors and certain diseases also causing telomere damage (86, 94). Patients with these disorders exhibit premature cellular senescence, telomere aberrations, or random telomere loss (86, 95), reducing the number of differentiated osteoblasts and osteocytes, thereby promoting osteoporosis. Telomeric alterations drive stem cell senescence: From molecular erosion to functional decline (see **Figure 1**).

### 3.2 Regulatory and Facilitating Mechanisms of MSCs Senescence

#### 3.2.1 Transcription and transcriptional regulation abnormalities in mscs senescence

In MSCs senescence, transcription and its regulatory network become widely dysregulated, encompassing both upstream epigenetic modifications that influence transcriptional activity and downstream disturbances in mRNA processing and modifications. Together, these alterations disrupt the stability and plasticity of gene expression in MSCs, progressively driving them into a senescent state.

During senescence, MSCs undergo epigenetic modifications, including histone modifications, DNA methylation, and chromatin remodeling, which are closely linked to transcriptional regulation (96, 97). Histone modifications regulate senescence by modulating the transcriptional activity of DNA regions associated with the cell cycle (98). Generally, histone acetylation and methylation promote transcription, while phosphorylation and ubiquitination tend to inhibit it (99). Defects in histone deacetylase upregulate the histone demethylase JMJD3 and indirectly downregulate polycomb group genes via the RB/E2F pathway, leading to p16^INK4A transcription activation and H3K27me3 demethylation, thereby promoting MSCs senescence (97, 100). Additionally, SIRT6 maintains genomic integrity and prevents cellular senescence by deacetylating H3K9, H3K18, and H3K56, regulating transcription factor recruitment and promoting repressive heterochromatin structures (31, 101). DNA methylation profiles are also associated with MSCs senescence. DNA methyltransferase-catalyzed DNA methylation typically suppresses transcription (31). Age-associated methylation changes, particularly H3K9me-promoted hypermethylation of p16^INK4A, are key features of epigenetic senescence in MSCs (31, 102). Furthermore, hypermethylation of key osteogenic transcription factors, such as Hox and Runx2, reduces their expression, impairing osteogenesis and accelerating osteoporosis (103, 104). Chromatin remodeling also plays a critical role in transcriptional regulation during senescence. For example, Brg1-mediated SWI/SNF chromatin remodeling maintains MSCs transcriptional activity, and its loss facilitates DNA methyltransferase recruitment to the Nanog promoter, repressing transcription and accelerating senescence (105). Notably, endogenous hormonal fluctuations—particularly the decline in estrogen levels during menopause—serve as important disruptors of the epigenetic regulatory network in MSCs (106). Studies have shown that estrogen, via its receptor ERα, interacts synergistically with the epigenetic regulator EZH2 to modulate the expression of adipogenic transcription factors in MSCs and to maintain H3K27me3 levels at their promoters (219). This interaction governs MSCs lineage commitment and functional homeostasis (219). In postmenopausal conditions, estrogen deficiency disrupts this regulation, contributing to senescence-associated phenotypic alterations in MSCs, impairing osteogenesis, and thereby accelerating osteoporosis progression (219).

mRNA processing and modifications are closely linked to MSCs senescence. The transcription rate of RNA polymerase II increases with age, but proper exon splicing depends on optimal transcription rates. Excessive transcription rates may lead to exon skipping and intron retention (107, 108). Since RNA polymerase II elongation is regulated by factors such as SPT5, PAF1C, SPT6, and SEC, targeting these elongation factors or increasing histone gene expression may represent novel strategies to delay senescence (109). Transcription factors also contribute to MSCs senescence. For instance, NF-κB activation triggers the SASP, promoting inflammation and accelerating senescence (110, 111). Downregulation of FOXO1 accelerates MSCs senescence and alters the transcriptome (112, 214). mRNA modifications, particularly m6A methylation, are critical in MSCs senescence. Enhanced m6A levels in senescent MSCs are mitigated by ALKBH5 through its m6A demethylation activity (113, 115). METTL3, an RNA-modifying enzyme, stabilizes MIS12 transcripts via m6A modification, attenuating MSCs senescence (115, 116). RNA-binding proteins, such as HuR, regulate mRNA stability through selective splicing, 5’-end capping, and 3’-end polyadenylation. HuR delays MSCs senescence by stabilizing SIRT1 mRNA, but its levels decline with senescence, impairing cellular function (117). Despite these insights, the precise mechanisms by which transcriptional dysregulation drives MSCs senescence remain unclear and warrant further investigation.

#### 3.2.2 Non-coding RNA-mediated post-transcriptional regulation in mscs senescence

Non-coding RNAs, including long non-coding RNAs (lncRNAs) and microRNAs (miRNAs) (2), play critical roles in cellular processes. During senescence, the miRNA expression profile of MSCs is markedly altered in response to oxidative stress, inflammatory microenvironments, epigenetic modifications, and metabolic dysregulation, thereby affecting cellular function by targeting key senescence-associated genes. Among these, miRNAs regulate aging by selectively binding to the 3’-untranslated region of target mRNAs, inhibiting translation or promoting mRNA degradation (118, 119). For instance, miR-504, miR-125b, miR-25, and miR-30d delay senescence by directly binding to p53 mRNA and suppressing p53 protein expression (120). Conversely, miR-192, miR-194 and miR-605 stabilize p53 by targeting MDM2, leading to cell cycle arrest and senescence induction (120). The cell cycle regulator p21 is targeted by multiple miRNAs, including the miR-106b family, miR-130b, and miR-302a, which inhibit p21 expression to maintain cell cycle progression and delay senescence (118). miR-195 promotes senescence by targeting SIRT1 and TERT, inhibiting telomere elongation and enhancing p53 signaling (121). Additionally, miR-486-5p accelerates MSCs senescence by binding to the 3’-untranslated region of the SIRT1 gene, suppressing osteogenic and adipogenic differentiation (30). In the context of p16^INK4a suppression, miR-24 inhibits its translation, maintaining cell cycle progression and delaying senescence (30, 122). Furthermore, miR-26b, miR-181a, miR-210, and miR-424 target polycomb repressive complex proteins (e.g., CBX7, EED, EZH2, and Suz12), inducing p16 upregulation and accelerating senescence (123). lncRNAs also regulate cellular senescence by competitively binding to miRNAs, modulating the translational efficiency of target mRNAs (124). For example, Xist lncRNA competitively binds to miR-19a-3p, inhibiting its activity and thereby impairing osteogenic differentiation of BMSCs while accelerating senescence (125). Similarly, lincRNA-p21, induced by p53, increases ROS levels and accelerates MSCs senescence by regulating p21 transcription and inhibiting the Wnt pathway (126). Moreover, during menopause, declining estrogen levels lead to increased oxidative stress and elevated pro-inflammatory cytokines such as TNF-α and IL-6. These inflammatory signals influence transcription factors that markedly reshape miRNA expression profiles, disrupting the balance between osteoblasts and osteoclasts. This imbalance results in reduced bone formation and enhanced bone resorption. For instance, the accumulation of ROS has been shown to upregulate miR-141, which suppresses the expression of osteogenic proteins and thereby exacerbates osteoporosis progression (127). In summary, the regulation of senescence-related genes such as p53, p21, SIRT1, and p16^INK4a by miRNAs and lncRNAs influences cell cycle progression, proliferation, differentiation, and senescence. Modulating the expression of these ncRNAs may provide novel strategies to delay MSCs senescence and enhance their therapeutic potential. Transcriptional regulation in MSCs senescence: epigenetic programming and mRNA processing networks (see **Figure 1**).

#### 3.2.3 Protein homeostasis imbalance in MSCs senescence

Protein homeostasis, the process of maintaining proper protein folding, function, and clearance of misfolded proteins, is critical for cellular health (128, 129). In MSCs, protein homeostasis imbalance is a key driver of aging. Protein homeostasis imbalance impairs MSCs function by promoting the accumulation of misfolded proteins and disrupting their degradation, which in turn triggers oxidative stress and inflammatory responses. These disturbances form a vicious cycle that ultimately accelerates cellular senescence. This imbalance arises from two major factors: diminished molecular chaperone function and impaired protein degradation systems. Molecular chaperones maintain protein homeostasis by assisting protein folding, preventing misfolding and aggregation, and promoting the degradation of damaged proteins (130). However, chaperone function declines with aging. For example, Hsp70 upregulation is regulated by HSF1, but reduced HSF1 activity in senescent cells limits chaperone protein expression (131, 132), leading to protein homeostasis imbalance (133). This imbalance compromises MSCs function and promotes senescence. The protein degradation systems, including the ubiquitin-proteasome system (UPS) and autophagy, also deteriorate with age. Senescence reduces UPS function, characterized by decreased proteasomal subunit expression, assembly defects, and reduced ubiquitination levels, primarily due to increased deubiquitinating enzyme activity and diminished ubiquitin-conjugating enzyme activity (134). UPS dysfunction impairs the clearance of misfolded proteins, leading to protein aggregation and functional disruption, thereby accelerating MSCs senescence (135, 136). Autophagy is significantly impaired in osteoporotic conditions (137). For instance, sustained activation of mTORC1, a major negative regulator of autophagy, inhibits autophagic activity and disrupts protein homeostasis (138, 139). Additionally, lysosomal pH, enzyme activity, and membrane fusion capacity decline with age, further impairing protein degradation and causing metabolic imbalance (140). In aged BMSCs, reduced expression of autophagy-related proteins (e.g., Atg7, Beclin1, and P62) hinders the clearance of dysfunctional mitochondria and damaged proteins, elevating ROS levels and increasing DNA damage. These changes accelerate BMSCs senescence and promote osteoporosis (141). Furthermore, the age-related decline in cellular antioxidant defenses increases ROS levels and reduces ATP synthesis (8). These factors not only damage protein structure and function but also inhibit protein degradation systems (142, 143), creating a vicious cycle of mitochondrial damage and ROS accumulation that further accelerates MSCs senescence (8). In summary, protein homeostasis imbalance drives MSCs senescence through multiple mechanisms, including impaired autophagy, UPS dysfunction, and oxidative stress, contributing to the development and progression of age-related diseases such as osteoporosis. Proteostatic control of aging: translational fidelity and protein quality surveillance (see **Figure 1**).

## 4 Innovative and effective therapies for osteoporosis

### 4.1 Stem cell therapy in osteoporosis treatment

Decreased osteogenic capacity triggered by senescence of BMSCs leads to decreased BMD (144, 145), and supplementation of MSCs and induction of bone tissue regeneration can effectively improve osteoporosis (146). Since Bab et al. first demonstrated the osteogenic potential of BMSCs in 1998 (147), stem cell therapy has shown promising results in various osteoporosis animal models. The therapeutic mechanisms include: 1. MSCs Nesting role: Adipose-derived stem cells injected via the tail vein can target bone tissues, and aspirin enhances this homing effect, improving bone loss in ovariectomized rats (148). CXCR4-transfected BMSCs increase vertebral bone density and biomechanical properties in rats (149), highlighting the role of MSCs homing in promoting osteogenesis. 2.Direct osteogenic differentiation: Overexpression of Mettl3 in BMSCs enhances osteogenic differentiation and prevents osteoporosis in ovariectomized mice, while its deficiency leads to reduced bone mass and bone marrow adiposity. Younger MSCs exhibit greater osteogenic capacity (150). 3. Regulation of osteoclast function: MSCs inhibit osteoclast differentiation by secreting osteoprotegerin (151). Additionally, BMSCs suppress osteoclastogenesis through WNT-1 expression, maintaining bone mass balance (215). 4. BMSCs promote angiogenesis, enhancing osteogenesis and bone repair: Zhang et al. demonstrated that BMSCs improve osteogenesis and repair bone defects in rats (218). Similarly, Jia et al. found that local injection of exosomes in a rat tibial bone defect model enhances endothelial cell migration, accelerates angiogenesis, and facilitates bone repair (154). However, direct evidence of MSCs-mediated bone repair through angiogenesis *in vivo* remains lacking and requires further investigation. In addition, utilizing stem cells as a novel source of osteoblasts and guiding their differentiation through the creation or exploitation of appropriate mechanical environments to promote bone formation and restore skeletal mechanostasis may represent an emerging strategy in stem cell-based therapy (155).

**Figure 2:**
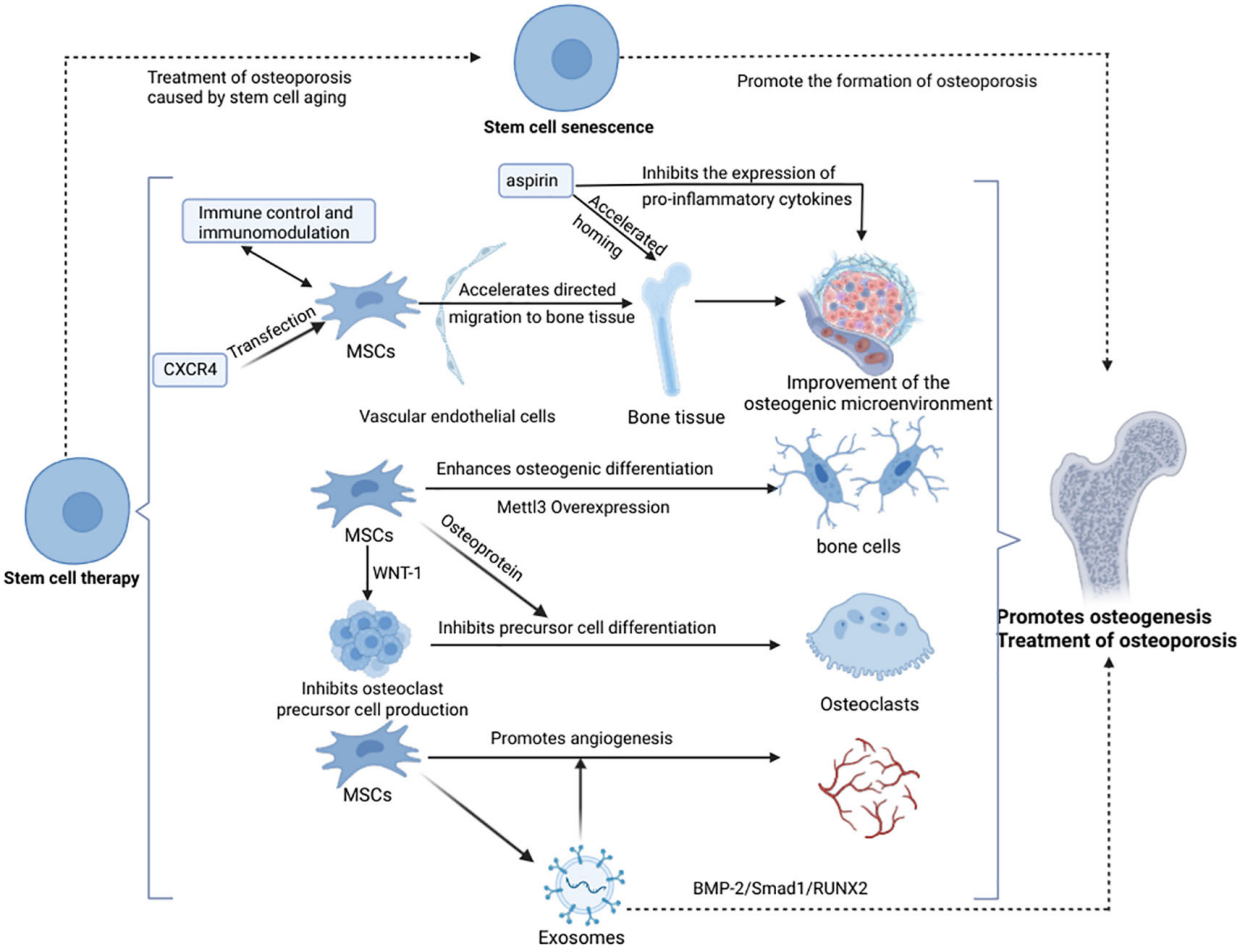
Stem cell therapy promotes osteogenesis and treats osteoporosis (Created in https://BioRender.com).

Despite advancements in clinical trials of stem cell therapies, limitations such as small sample sizes, short study durations, and inconclusive results persist. Therefore, additional clinical data are needed to confirm the efficacy of stem cell therapy in osteoporosis and optimize treatment protocols for clinical translation (see **Figure 2**).

### 4.2 Research progress on drug and small molecule therapies targeting the mechanism of MSCs senescence in the treatment of osteoporosis

Modulating aging-related signaling pathways, restoring impaired cellular function, and selectively eliminating senescent cells through targeted drugs and small molecules have emerged as promising strategies to reverse MSCs senescence, representing a current research hotspot in the treatment of osteoporosis. Among these, SASP inhibitors mitigate the aging process of MSCs by reducing the secretion of inflammatory cytokines and SASP factors. Compounds such as rapamycin, melatonin, resveratrol, metformin, and ferulic acid have demonstrated notable SASP-inhibitory effects, primarily through suppression of the mTOR signaling pathway or activation of AMPK signaling. These mechanisms help to attenuate chronic inflammation during MSCs senescence, thereby showing therapeutic potential in age-related osteoporosis (96, 141, 152). The targets and mechanisms of these SASP inhibitors are summarized in **Table 1** (see **Table 1**). In addition to SASP inhibition, a group of emerging therapeutic strategies—including senolytic agents, small molecule-targeting compounds, and antioxidants—have shown broad potential in delaying MSCs senescence and promoting bone regeneration. Senolytic agents, such as dasatinib and quercetin, can selectively eliminate senescent MSCs accumulated in tissues, thereby restoring regenerative capacity and slowing the progression of osteoporosis (96, 168, 169). Furthermore, small molecule-targeting compounds such as fibroblast growth factor 21 and insulin-like growth factor 1 can activate downstream signaling pathways to enhance MSCs proliferation, differentiation, and resistance to apoptosis. In parallel, antioxidant agents such as nicotinamide and its precursor nicotinamide adenine dinucleotide mitigate ROS generation and oxidative stress, thereby slowing MSCs aging and contributing positively to osteoporosis therapy (170, 171). Although some of these agents were identified decades ago, their potential in reversing MSCs senescence and treating osteoporosis has only recently been elucidated. A comprehensive summary of these interventions is presented in **Table 2** (see **Table 2**). While many of the aforementioned agents remain in the preclinical or early research phase, accumulating evidence supports their therapeutic promise in managing osteoporosis. Future studies are warranted to further validate their efficacy and safety, with the aim of developing novel treatment options for patients with aging-related bone disorders.

**Table 1:** Targets and mechanisms of SASP inhibitors in reversing MSCs aging and treating osteoporosis.

Drug	Mechanism of action	Impact on MSCs Aging	Effect on osteoporosis	References
Rapamycin	1. Inhibits mTOR signaling 2. Induces autophagy and reduces ROS levels	Reduces SASP-related inflammation by decreasing SASP factor production; Enhances senescent MSCs functionality	Increases bone density;Enhances osteogenesis; Suppresses adipocyte formation; Stimulates cell proliferation	(96, 156, 157)
Melatonin	1.Clears p53/ERK/p38 pathway 2. Reduces ROS accumulation 3. Activates AMPK signaling, upregulating FOXO3a and Runx2	Protects MSCs against oxidative stress: Decreases secretion of SASP factors; Enhances osteogenic/chondrogenic differentiation capacity	Promotes bone formation;	(158, 96) (159, 152)
Metformin	1. Activates AMPK signaling and downregulates miR-34a-3p 2. Inhibits mTOR phosphorylation 3. Decreases ROS levels 4. Modulates miR-181a-5p/PAI axis	Mitigates oxidative stress,delaying MSCs aging and reducing DNA damage; Suppresses SASP factors secretion	Shows therapeutic potential for osteoporosis	(160–164)
Resveratrol	1. Activates SIRT1 modulating oxidative stress and inflammation 2. Modulates mitochondrial gene transcription 3. Preserves telomere integrity	Delays MSCs senescence	Enhances bone microarchitecture	(96, 165, 166)
Ferulic Acid	1. Inhibits NF-kB signaling pathway 2. Decreases ROS generation	Attenuates oxidative stress damage; Suppresses SASP factor secretion	Stimulates osteoblast proliferation	(167)

**Table 2:** Advances in senolytics and other small molecule-targeting agents for reversing MSCs senescence and treating osteoporosis.

Druss/small molecules	Mechanism of action	Impact on MSCs Aging	Effect on osteoporosis	References
Senolytic Drugs				
Dasatinib, Quercetin, Fisetin	Inhibits PI3K/Akt signaling pathway	Selectively eliminates senescent cells;Attenuates chronic inflammation	Enhances bone mineral density	(96, 153, 169, 172, 173, 216)
Curcumin	Upregulates TAZ expression	Augments osteogenic potential of bone marrow-derived MSCs	Enhances trabecular bone microarchitecture; Promotes MSCs osteolineage commitment	(168)
Small Molecule-Targeting Agents				
Human embryonic stem cell-derived small EVs	Activates Wnt/Sirtuin/AMPK/PTEN signaling and other pro-regenerative pathways	Upregulates anti-aging genesrestore the function of aging MSCs	Enhances bone microarchitecture Reduces osteoporosis severity	(174, 175)
Fibroblast growth factor 21	Activates the AMPK signaling pathway	Improves the quality and quantity of MSCs, delays the aging of MSCs	Improves bone quality; Reduces osteoporosis.	(175, 176)
Insulin-like growth factor 1	Activates the p13k/Akt pathway	Promotes the proliferation and osteogenic differentiation of MSCs	Improves bone density	(114, 177)
Others				
Nicotinamide	Reduces ROS production	Reduces oxidative stress: Slows the aging of MSCs and DNA damage	Improves bone quality; Reduces osteoporosis	(88, 170)
Ascorbic acid	1. Inhibits AKT/mTOR signaling 2. Reduces ROS production	Reduces oxidative stress: Improves MSCs function	Promotes bone formation	(97, 171)

### 4.3 The application of novel therapies using hydrogels and extracellular vesicles in the treatment of osteoporosis

The development of osteoporosis is closely linked to the disruption of bone tissue structure. Actively improving the microenvironment and tissue structure creates more favorable conditions for stem cell survival. In bone tissue engineering, hydrogels have been employed as scaffolds to address osteoporotic bone defects, enhance the bone microenvironment, promote osteogenic differentiation of MSCs, and delay cellular aging. Notably, stimulus-responsive hydrogels, which can modulate mechanical properties, shape, and drug release in response to triggers such as temperature, pH, electromagnetic radiation, magnetic fields, or biological factors, have become a prominent focus in bone-enabling research (178–180). For example, Ye et al. developed a thermo-responsive injectable hydrogel (MnO_2_@Pol/HA) that supports osteogenic differentiation of BMSCs by scavenging ROS and modulating macrophage polarization (181). Tang et al. designed a dual-network hydrogel (GelMA/ALN-OSA) that responds to pH changes to maintain stable drug concentrations and promote bone regeneration (182). Zhou et al. developed an electrochemical deposition-constructed hydrogel (Mg@PEG-PLGA) that scavenges ROS by releasing H_2_ through hydrolysis reactions, improves the bone microenvironment, and promotes osteogenic differentiation of MSCs (183). However, further optimization of the hydrogel’s biocompatibility, mechanical properties, and biodegradability is necessary to ensure synergistic effects with the bone regeneration process (184).

Recent studies have also highlighted the potential of EVs, including exosomes, due to their unique nanostructures, stable drug-carrying capacity, and excellent biocompatibility. EVs can improve the bone microenvironment, delay MSCs aging, and have become a key area of interest in osteoporosis research. Liu et al. used synthetic biology to integrate BMP-2 and CXCR4 onto the surface of bacterial EVs to deliver BMP-2, activating osteogenic signaling and significantly enhancing bone density and strength while inhibiting adipogenesis, thereby improving osteoporosis (112). EVs from various sources have demonstrated promising applications in osteoporosis treatment through multiple mechanisms (see **Table 3**). Additionally, the combination of EVs with hydrogels has shown significant potential in bone regeneration. Ding et al. developed a gelatin/ECM composite scaffold loaded with apoptotic vesicles derived from adipose-derived MSCs under hypoxic conditions. This scaffold significantly promoted osteochondral defect repair in rat osteoarthritis by enhancing stem cell proliferation, migration, and cartilage-forming differentiation, as well as promoting macrophage M2 polarization (193). Guo et al. developed a GEL-OCS/MBGN composite hydrogel loaded with EVs, which significantly promoted bone defect repair in rats by enhancing osteogenic differentiation of BMSCs through modulation of the miR-19b/WWP1 axis (194). Despite these advancements, further in-depth studies and clinical validation are needed to fully realize the potential of EVs and hydrogels in osteoporosis therapy.

**Table 3:** Treatment of osteoporosis with EVs from different sources.

Cel Slource	Mechanism	Impact on MSCs Aging	Effect on Osteoporosis
BMSCs (35, 185)	1. Contains microRNA-122-5p 2. Regulates the USP7/YAP1/β-catenin axis 3.Modulates Wnt/β-catenin, Hippo, and PI3K/Akt signaling pathways 4. Enhances osteogenic protein expression	Reduces senescence markers; Delays MSCs aging; Decreases oxidative stress and inflammation	Reduces bone loss; Increases bone mass and strength
Urine-Derived MSCs (186, 187)	1. Enriched in CTHRC1 2. Contains abundant osteoprotegerin protein 3. Promotes angiogenesis	Improves the microenvironment to promote bone differentiation	Improves bone quality; Promotes bone formation
Adipose-Derived MSCs (188)	1. Targets genes related to the RANKL-RANK pathway 2. Enriched in osteoprotegerin	Promotes the homing of MSCs and osteogenic differentiation	Promotes bone remodeling
Amniotic Fluid-Derived MSCs (189)	1. Contains antioxidant enzymes 2. Enhances the expression of osteogenesis-related proteins 3. Activates SIRT1 and Nrf2 pathways	Improves the slowdown of MSCs aging and DNA damage	Promotes bone formation; Delays local bone loss
Osteoclasts (190)	1. Regulates RANKL-RANK signaling 2. Targets ARHGAP1	Promotes the osteogenic differentiation of MSCs and improves the bone microenvironment	Promotes osteogenesis; Enhances bone quality
Bacteria (191, 192)	1. Regulates the activity of pro-inflammatory cytokines and immune cells 2. Regulates proteins related to calcium absorption pathways and alter the intestinal microenvironment	Improves the bone microenvironment	Prevents bone loss; Enhances bone quality

## 5 Conclusion and outlook

### 5.1 The key connection and influence mechanism between MSCs aging and osteoporosis

The aging of MSCs plays a critical role in the pathogenesis of osteoporosis. Studies have demonstrated that the osteogenic capacity and self-renewal potential of MSCs decline significantly with age, a phenomenon closely associated with reduced expression of osteogenic markers, elevated oxidative stress levels, and increased inflammatory responses (195–197). Specifically, DNA damage accumulated in MSCs during aging activates cell cycle regulatory pathways, particularly the p53-p21 pathway, leading to cell cycle arrest (198). This arrest not only impedes normal cell proliferation and differentiation but also directly affects the repair and regenerative capacity of bone tissue, thereby accelerating the progression of osteoporosis (199). Therefore, a deeper understanding of the specific mechanisms underlying MSCs senescence can help elucidate the pathophysiological characteristics of osteoporosis and provide more targeted intervention strategies (200).

### 5.2 Prevention and treatment of osteoporosis caused by MSCs aging

Given the strong link between MSCs aging and osteoporosis, interventions targeting the aging process of MSCs are of paramount importance. Existing studies have shown that lifestyle modifications (e.g., moderate exercise, a balanced diet, smoking cessation, and alcohol restriction) and the development of novel drugs can effectively delay MSCs aging, thereby reducing the risk of osteoporosis (201, 202). For instance, physical inactivity has been associated with a significant decline in BMD, and studies have found that older women who engage in regular exercise exhibit significantly higher BMD compared to those who are sedentary (203). Additionally, smoking interferes with calcium absorption and increases oxidative stress, damaging bone cells and accelerating bone loss (204, 205). Excessive alcohol consumption not only inhibits osteoblast activity but may also indirectly affect bone metabolism through liver damage (206, 207). Thus, improving lifestyle habits not only helps delay MSCs aging but also promotes overall bone health and slows the progression of osteoporosis. Therapeutic strategies targeting oxidative stress and inflammation may also offer new avenues for osteoporosis prevention and treatment (127, 196, 208).

### 5.3 Directions for future research

Future research should focus on exploring the mechanisms of MSCs aging and its role in osteoporosis at the cellular and molecular levels. In particular, in-depth studies on the interactions between cell cycle regulation, oxidative stress, and inflammatory responses are needed to identify potential intervention targets (209). Additionally, the development of novel drugs and intervention strategies, such as gene editing technologies or small molecule compounds, may become effective means to delay MSCs aging. In terms of clinical applications, the focus should be on evaluating the efficacy and safety of these interventions in diverse populations (e.g., the elderly and individuals at high risk for osteoporosis), aiming to provide more effective solutions for osteoporosis prevention and treatment (210–212). These studies will not only deepen our understanding of the impact of aging on bone health but also provide new theoretical foundations and practical guidance for the prevention and treatment of related diseases.
